# Entecavir combined with furin inhibitor simultaneously reduces hepatitis B virus replication and e antigen secretion

**DOI:** 10.1186/1743-422X-11-165

**Published:** 2014-09-16

**Authors:** Hui Y Yang, Nai Q Zheng, Dong M Li, Lin Gu, Xiao M Peng

**Affiliations:** Hepatology Laboratory, the Hospital for Liver Disease, Sun Yat-Sen University, 600 Tianhe Road, Guangzhou, 510630 China; Department of Infectious Diseases, the Third Affiliated Hospital, Sun Yat-Sen University, Guangzhou, China; Liver Disease Key Laboratory of Guangdong Province, Guangzhou, China

**Keywords:** Hepatitis B virus, Viral replication, Hepatitis B e antigen, Proprotein convertase, Furin, Antiviral therapy

## Abstract

**Background:**

The antiviral therapy of chronic hepatitis B virus (HBV) infection pursues the dual goals, virological response (undetectable serum HBV DNA) and hepatitis B e antigen (HBeAg) serological response (serum HBeAg loss/seroconversion). It is relatively difficult, however, to realize the serological response, especially for nucleotide/nucleoside analogs. Furin, a proprotein convertase, is involved in HBeAg maturation. The suppression of furin using inhibitors accordingly reduces HBeAg secretion, but possibly enhances HBV replication. For these reasons, the strategy based on the combination of nucleoside analog entecavir (ETV) and furin inhibitors to inhibit HBV replication and HBeAg secretion simultaneously were studied here.

**Methods:**

The suppression of furin was performed using inhibitors decanoyl-RVKR-chloromethylketone (CMK) and hexa-D-arginine (D6R) or the expression of furin inhibitory prosegment. The influence of furin suppression on HBV replication and the effect of CMK combined with nucleoside analog entecavir (ETV) on HBV replication and HBeAg secretion was investigated in HepG2.2.15 cells. HBeAg level in media was detected using enzyme-linked immunosorbent assay. Intracellular viral antigens and HBV DNA were detected using Western and Southern blotting analyses, respectively.

**Results:**

CMK, D6R and the expression of inhibitory prosegment all significantly reduced HBeAg secretion, but only CMK enhance HBV replication. Concordantly, only CMK post-transcriptionally accumulated cytosolic HBV replication-essential hepatitis B core antigen (HBcAg). The HBcAg-accumulating effect of CMK was further found to be resulted from its redundant inhibitory effect on the trypsin-like activity of cellular proteasomes that are responsible for HBcAg degradation. Moreover, the viral replication-enhancing effect of CMK was abrogated by ETV and ETV combined with CMK reduced HBV replication and HBeAg secretion simultaneously.

**Conclusion:**

The suppression of furin itself does not enhance HBV replication. Nucleotide/nucleoside analogs combined with furin inhibitors may be a potential easy way to realize the dual goals of the antiviral therapy for chronic hepatitis B in the future.

## Background

Hepatitis B virus (HBV) infections annually cause 1 million of deaths worldwide [1, 2]. Antiviral therapy is an important way to improve the prognoses of these victims. Hepatitis B surface antigen (HBsAg) loss or seroconversion is thought as a perfect endpoint of current antiviral therapy. However, hepatitis B e antigen (HBeAg) seroconversion (HBeAg serological response) and undetectable HBV DNA (virological response) are common goals to be pursued in clinical practice since on-treatment HBsAg loss or seroconversion is difficult to realize, but it would automatically occurs in patients from years to decades after HBeAg seroconversion. HBeAg is a well-known immune-toleragen [3]. Its persistence is an independent risk factor for hepatocellular carcinoma and is associated with a lower survival rate among cirrhotic patients [4, 5]. In contrast, HBeAg seroconversion is thought to be important in establishing a benign prognosis [6, 7]. Compared with virological response only, virological response plus HBeAg serological response have a low relapse relate while off treatment of current antiviral therapy [7]. Though those patients with HBeAg seroconversion (HBeAg-negative chronic hepatitis B) due to infection of HBeAg-defective variants also have poor prognoses, the significance of HBeAg to HBV infection is not minimized since these variants rarely cause a *de novo* chronic infection [8], implying that HBeAg loss may be helpful for termination of chronic HBV infection. Therefore, early antiviral intervention in HBeAg-positive chronic hepatitis B may benefit all patients. In addition, early therapeutic intervention is helpful to reduce the risks for long-term complications while on-treatment [9, 10]. However, current antiviral options including recombinant interferon and nucleoside/nucleoside analogs cannot rapidly and economically realize the dual goals of the antiviral therapy. For example, nucleoside analog entecavir (ETV) blocks HBV replication rapidly, but induce HBeAg seroconversion unpredictably. For these reasons, ETV combined with some direct HBeAg secretion-inhibitory measures seems a strategy to improve the current antiviral therapy of chronic hepatitis B.

HBeAg is encoded by the C open reading frame of the viral genome. This frame also encodes viral core protein (also called hepatitis B core antigen, HBcAg, 21 kDa). Compared with HBcAg, the initial peptide of HBeAg has an extra precore region consisting of a 19-amino acid signal peptide that directs the nascent peptide into the secretory pathway. After the signal peptide is removed in the lumen of the endoplasmic reticulum, the HBeAg precursor is generated and transported to the *trans*-Golgi network. The HBeAg precursor (pre-HBe, 22 kDa) is further cleaved by proprotein convertase furin in the arginine-rich domains of the C-terminus to generate the mature HBeAg (17 ~ 20 kDa) [11, 12]. Furin belongs to the subtilisin/kexin-like serine protease family. It is responsible for the majority of proprotein processing, and thus not only plays critical roles in normal cell growth and differentiation, but is also involved in many disease states, such as Alzheimer’s disease, tumoriogenesis, and infections [13]. Our previous studies have shown that persistent HBV infection prefers to occur in patients carrying highly active genotypes of furin and furin inhibitors, decanoyl-RVKR-chloromethylketone (CMK) and hexa-D-arginine (D6R), reduce HBeAg secretion without interfering with cellular protein secretion in HepG2.2.15 cells [14, 15]. In addition, HBeAg reduction resulted from furin inhibition leads to increase in cell surface expression of immune-promoting pre-HBe [15], which is different from HBeAg reduction or loss caused by infection of HBeAg-defective variants, suggesting that furin inhibition may have less risk to let the infection develop into poorly prognostic HBeAg-negative chronic hepatitis B. Though it is not a traditional antiviral strategy, the direct inhibition of HBeAg secretion mediated by the immune-regulation effects may be helpful for the treatment of chronic HBV infection. Thus, the suppression of furin may be a promising candidate way to improve HBeAg serological response to ETV by inhibiting HBeAg secretion directly.

CMK and D6R are small synthetic furin inhibitors that are suitable for the clinical purpose. CMK is a referential furin inhibitor in many laboratories. It is more effective than D6R in reduction of HBeAg secretion [15]. However, CMK and a mutation (T147A) adjacent to a putative furin recognition site (^151^RRGR^154^) in HBcAg of HBeAg-defective variant (carrying the precore stop mutation, G1896A) have been found to enhance HBV replication [15, 16]. In the current study, we wonder whether the suppression of furin by other measures enhances HBV replication and the combination with ETV inhibits HBV replication and HBeAg secretion simultaneously. As results, furin suppressed by measures other than CMK did not enhance HBV replication. CMK was found to enhance HBV replication by inhibiting the trypsin-like (TL) activity of proteasomes. The viral replication-enhancing effect of CMK was abrogated by ETV. ETV combined with CMK could simultaneously reduce HBV replication and HBeAg secretion. These findings highlight a novel approach to improve the antiviral therapy for chronic HBV infections.

## Results

### Furin suppressed by measures other than CMK does not enhance HBV replication

**Figure 1:**
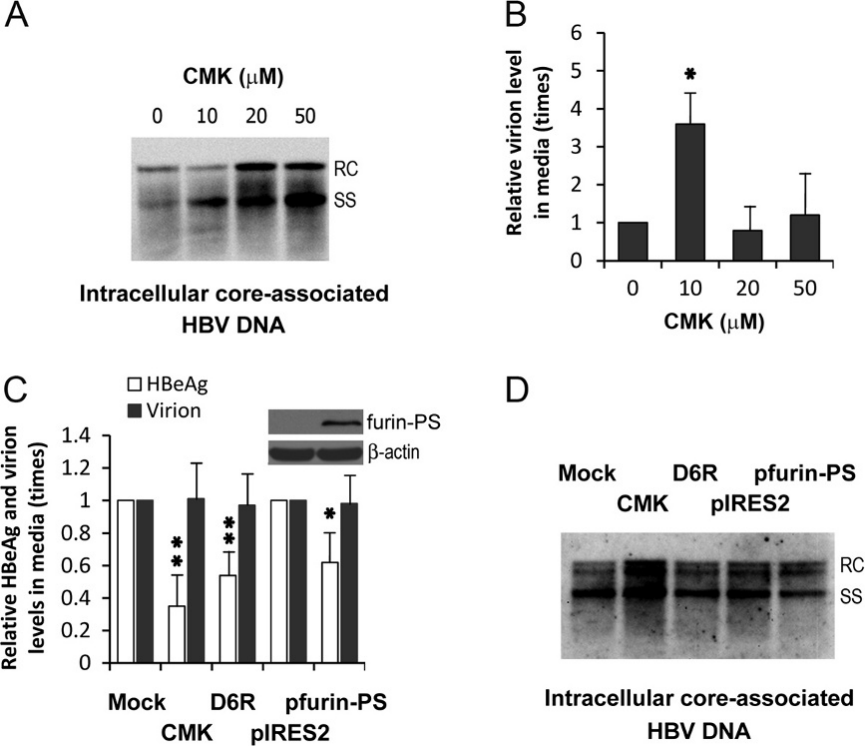
**The influences of furin suppression on HBV replication.** HepG2.2.15 cells were regularly cultivated and treated with and without furin inhibitors (20 μmol/L CMK and 100 μmol/L D6R unless marked particularly), or transfected with empty pIRES2-EGFP vector (pIRES2) or furin inhibitory prosegment-expressing recombinant vector (pfurin-PS). Intracellular core-associated HBV DNA was detected using Southern blot analysis. Relaxed circular (RC) HBV DNA was the HBV genome, and single-stranded linear (SS) HBV DNA was the replication intermediates. Virion-related HBV DNA in culture medium was quantified using real-time fluorescent PCR. HBeAg was detected using enzyme-linked immunosorbent assay. Furin inhibitory prosegment was detected using Western blot analysis. **A**: CMK increased intracellular core-associated HBV DNA. **B**: CMK increased or unaffected the level of HBV virions in the media. *P <0.05. **C**: D6R and the expression of furin inhibitory prosegment inhibited HBeAg secretion. *P < 0.05 and **P < 0.01. **D**: D6R and the expression of furin inhibitory prosegment did not enhance HBV replication.

A strain of HBeAg-defective variant (G1896A) harboring additional mutations, T147A and V149I, adjacent to a putative furin recognition site in the C-terminus of HBcAg, has higher replication efficiency than the conventional variant. CMK can enhance the replication of the latter variant to the level of the former variant [16]. Here, the influence of CMK on the replication of wild-type HBV was firstly tested. When HepG2.2.15, a wild-type HBV-producing cell line, was used in this study, CMK was found to increase the level of intracellular core-associated HBV DNA in a dose-dependent manner (Figure 1A). CMK reduces HBsAg secretion at high concentrations (15), perhaps by interfering with furin proteolyzation of HBV large surface antigen pre-S1 that simultaneously affect HBV virion release, implying that CMK at high concentrations may also interfere with HBV virion release; however, the enhancements based on the increased level of intracellular core-associated HBV DNA were not resulted from the limitation to virion release since the virion level in media did not significantly decrease (Figure 1B), and were neither related to the influence of CMK on cell viability. Though furin down-regulation using small interfering RNA has not been found to enhance HBV replication [16], we further tested whether HBV replication is enhanced by D6R and the expression of furin inhibitory prosegment that has been successfully used to inhibit furin activity [17]. As results, they both significantly reduced HBeAg secretion; however, they did not influence HBV replication and virion release (Figure 1C and D). The expression of furin inhibitory prosegment was successful (Figure 1C, top right corner).

### CMK accumulates cytosolic HBcAg and pre-HBe

**Figure 2:**
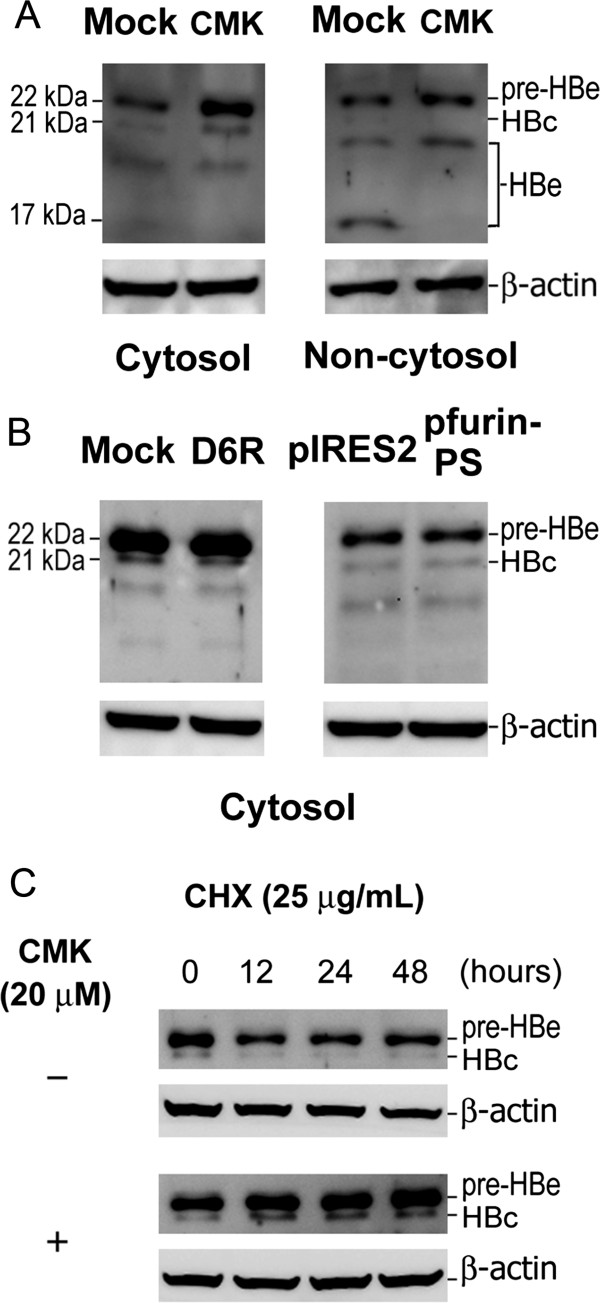
**The influences of furin suppression on the cellular expression of HBcAg and pre-HBe.** HepG2.2.15 cells were regularly cultivated and treated with or without furin inhibitors (20 μmol/L CMK and 100 μmol/L D6R), or transfected with empty pIRES2-EGFP vector (pIRES2) or furin inhibitory prosegment-expressing recombinant vector (pfurin-PS). HBcAg (HBc, 21 kDa), pre-HBe (22 kDa) and HBeAg (HBe, 17 ~ 20 kDa) in cytosol and non-cytosol were detected using Western blot analysis. **A**: CMK inhibited HBeAg maturation in non-cytosol and accumulated HBcAg and pre-HBe in cytosol. **B**: D6R and the expression of furin inhibitory prosegment did not accumulate cytosolic HBcAg and pre-HBe. **C**: The turnover rates of both HBcAg and pre-HBe in cytosol were down-regulated by CMK when new protein synthesis was halted by cycloheximide (CHX).

The fact that only CMK enhances HBV replication suggests that CMK may enhance HBV replication by furin-unrelated mechanisms. CMK has been found to accumulate intracellular HBcAg in the cells transfected with HBeAg-defective variants of HBV [16], and the level of intracellular HBcAg usually correlates the level of HBV replication [18, 19], suggest that HBcAg accumulation may be responsible for HBV replication enhancement. In this study, the effect of CMK was investigated in HBeAg-competent HepG2.2.15 cells. HBcAg in this setting would cross-react with pre-HBe and HBeAg during Western blot analysis. Since the critical processes of HBV replication, such as capsid assembly, pregenomic RNA encapsidation and reverse transcription, occur in cytosol, and HBeAg matures in non-cytosol (endoplasmic reticulum and *trans*-Golgi network) of hepatocytes, this study was performed after the cytosolic and non-cytosolic proteins were separately extracted as reported [20]. As results, CMK was found to accumulate HBcAg in cytosol and to restrain the HBeAg maturation evidenced by pre-HBe increase and HBeAg decrease in non-cytosol (Figure 2A), supporting it is the cytosolic accumulation of HBcAg that correlates with HBV replication enhancement. This notion was further supported by the findings that D6R and the expression of furin inhibitory prosegment without HBV replication-enhancing capacity did not accumulate cytosolic HBcAg (Figure 2B).

### CMK blocked the degradation of cytosolic HBcAg and pre-HBe

Besides cytosolic HBcAg, pre-HBe in both cytosol and non-cytosol was accumulated by CMK (Figure 2A). Our previous study has shown that pre-HBe accumulated by furin inhibition is subsequently expressed on cell surface rather than retro-translocated into cytosol [15]. Thus, the above findings, together with the similarity of HBcAg and pre-HBe in primary structure, suggest that CMK possibly interfere with the degradation of these antigens in cytosol. Indeed, the turnover rates of both HBcAg and pre-HBe were decreased by CMK when cellular protein biosynthesis was blocked by cycloheximide (Figure 2C).

### CMK selectively inhibited the TL activity of proteasomes

**Figure 3:**
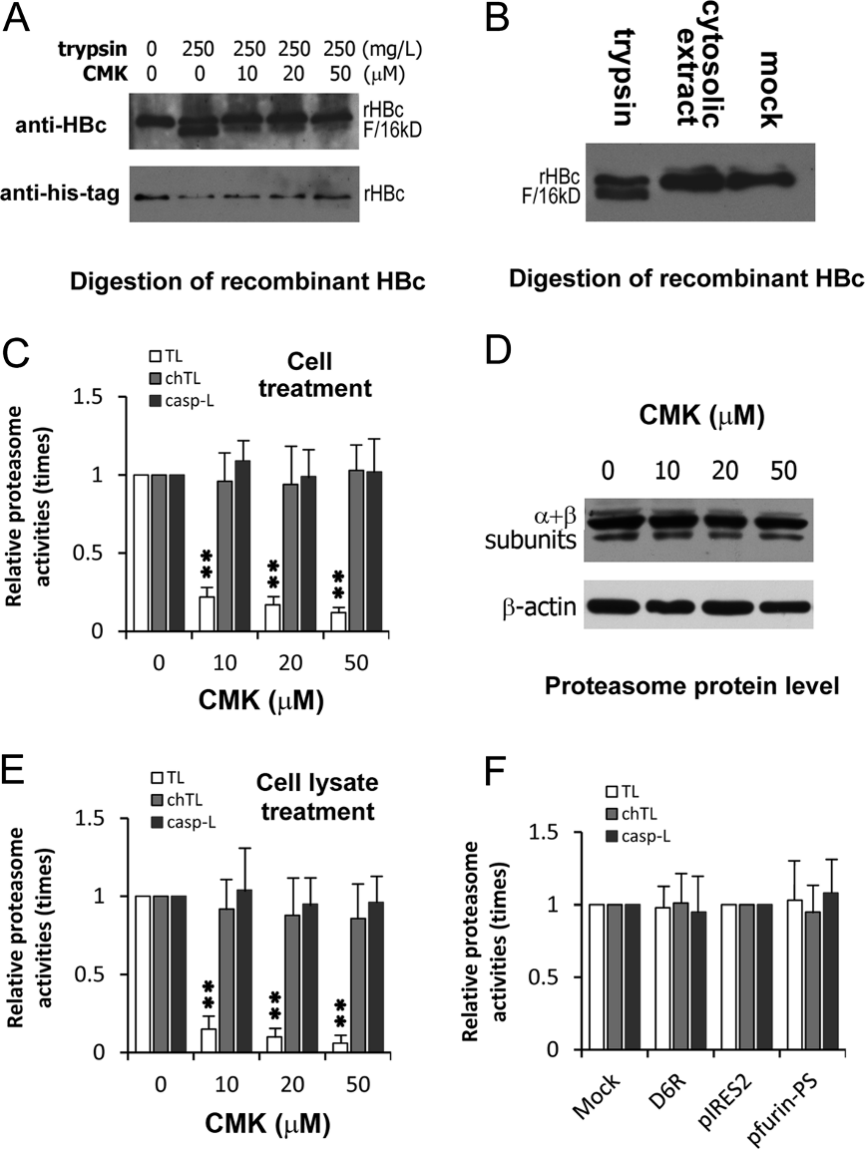
**The mechanism for CMK to accumulate cytosolic HBcAg and pre-HBe.** Recombinant HBcAg with 6 × his-tag in the C-terminus (rHBc) was digested by trypsin, and the products were demonstrated using Western blot analysis with either a polyclonal antibody to HBcAg (anti-core) or monoclonal antibody to 6 × his-tag (anti-his-tag). Proteasome activities were demonstrated using a commercial system, and results were expressed as times of the protease activities of the treatment groups over those of the control group. Columns and vertical bars represented the mean ± SD of three separate experiments. *P < 0.05 and **P < 0.01. **A**: CMK inhibited trypsin to digest rHBc into truncated fragments of 16 kDa (F/16kD). The truncated rHBc lost the 6 × his-tag. **B**: rHBc was not digested by the cytosolic extract from HepG2 cells. **C**: CMK selectively inhibited the TL activity when cells were treated for 2 hours. **D**: CMK did not affect the α and β subunits of proteasomes (α + β/subunits) in protein levels when compared with the housekeeping gene (β-actin). **E**: CMK selectively inhibited the TL activity when cell lysates were treated for 2 hours. **F**: D6R (100 μmol/L) and the prosegment expression did not affect the activities of proteasomes.

Furin and other proprotein convertases exist in the non-cytosolic fraction (e.g., the *trans*-Golgi network), supporting that these proteases are absolutely irrelevant to cytosolic HBcAg and pre-HBe degradations. In fact, cytosolic HBcAg and pre-HBe have been reported to be degraded in cellular ubiquitin-proteasome system [19–21]. However, the accumulation of truncated HBcAg by proteasome inhibitors [21] suggests that cytosolic HBcAg and pre-HBe are partially proteolyzed by some cellular proteases prior to proteasomal degradation. Cellular HBcAg existing as dimers in virion, nucleocapsids or capsids is usually resistant to proteases except for the protease-sensitive hinge (E^145^-D^153^, R^150^) between the capsid assembly-competent and pregenomic RNA encapsidation-competent domains of HBcAg [22]. Since that hinge is sensitive to trypsin and both furin and trypsin belong to the serine protease superfamily, the inhibitory effect of CMK on trypsin was studied *in vitro*. When recombinant HBcAg (156 amino acids) with a 6 × his-tag, 6 amino acids adjacent to R^150^, in the C-terminus was digested by trypsin, a truncated HBcAg of 16 kDa was generated, and CMK strongly blocked the digestion process (Figure 3A). The truncated HBcAg lacked of 6 × his-tag and theoretically had 150 amino acid residues based on its molecular weight, suggesting that the cleavage indeed occurred in the hinge.

Since cytosolic HBcAg and pre-HBe are inaccessible to trypsin in hepatocytes, their degradations could correlate with some cellular TL proteases, for example, hepsin, a small cellular TL protease. This protease primarily functions on the surface of hepatocytes, but some cytoplasmic form reportedly exists [23]. Unexpectedly, recombinant HBcAg was not digested by the cytosolic extract from HepG2 cells (parental cells of HepG2.2.15) (Figure 3B), suggesting that HBcAg and pre-HBe were directly degraded in the ubiquitin-proteasome system and that CMK could be capable of inhibiting the TL activity of proteasomes. Indeed, CMK selectively inhibited the TL activity without the changes in the protein level of proteasome subunits (Figure 3C and D), and even the selective inhibition was observed when the inhibitor was added to cell lysates (Figure 3E). On the other hand, D6R and the expression of furin inhibitory prosegment without HBcAg-accumulating capacity did not significantly affected the TL activity of proteasomes (Figure 3 F). Together, CMK directly and selectively inhibited the TL activity of proteasomes, which resulted in HBcAg accumulation and HBV replication enhancement.

### ETV abrogates the HBV replication-enhancing effect of CMK

**Figure 4:**
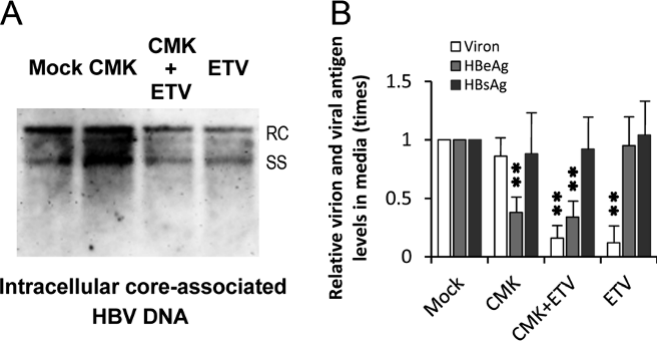
**Influences of ETV combined with CMK on the HBV replication and HBeAg secretion.** HepG2.2.15 cells were regularly cultivated and treated with or without CMK (20 μmol/L) and/or ETV (30 nmol/L). Intracellular core-associated HBV DNA was detected using Southern blot analysis. Relaxed circular (RC) HBV DNA was the HBV genome, and single-stranded linear (SS) HBV DNA was the replication intermediates. Virion-related HBV DNA in culture medium was quantified using real-time fluorescent PCR. HBeAg and HBsAg were detected using enzyme-linked immunosorbent assay. **A**: ETV abrogated the HBV replication-enhancing effect of CMK. **B**: ETV combined with CMK reduced HBV replication and HBeAg secretion simultaneously. **P < 0.01.

Although D6R and the expression of furin inhibitory prosegment both successfully reduced HBeAg secretion, D6R was less effective than CMK (Figure 1), perhaps due to the poor permeability [24], and the expression of the inhibitory prosegment depends on gene transfection techniques that their widespread uses are hindered by concerns over the safety *in vivo*. For these reasons, how to abrogate the HBV replication-enhancing effect of CMK was further tested in this study. ETV is one of the most effective nucleotide/nucleoside analogs that have been extensively used [25, 26]. Here, we found that ETV not only completely abrogated the enhancing effect of CMK, but also its HBV replication-inhibitory effect was not significantly affected by CMK (Figure 4A).

### ETV combined with CMK simultaneously reduces HBV replication and HBeAg secretion

ETV was effective in inhibiting HBV replication in HepG2.2.15 cells; however, it hardly affected HBeAg and HBsAg secretions (Figure 4B), which is concordant with its satisfactory virological response and dissatisfactory HBeAg serological response in clinical practice [27, 28]. On the other hand, CMK significantly inhibited HBeAg secretion, but enhanced HBV replication (Figure 1). In order to realize the dual goals of the antiviral therapy, the potential antiviral effects of ETV combined with CMK were further tested in HepG2.2.15 cells. The results clearly showed that ETV combined with CMK inhibited virion release and HBeAg secretion simultaneously though their inhibition efficiencies were comparable with those of mono-treatments (Figure 4B). Together with reducing intracellular core-associated HBV DNA (Figure 4A), the combination strategy indeed realized the simultaneous reduction of HBV replication and HBeAg secretion.

## Discussion

The current antiviral therapy of chronic HBV infection pursues the dual goals, virological and HBeAg serological responses; however, the later seems difficult to realize. Our previous study has shown that furin activity correlated with the outcome of HBV infection and furin inhibitors can significantly reduce HBeAg secretion without interference with the secretion of cellular secretory proteins [14, 15]. Unfortunately, CMK, the more effective inhibitor, enhances HBV replication [15, 16]. In this study, the suppressions of furin using D6R and the expression of furin inhibitory prosegment were not found to enhance HBV replication and HBV replication enhanced by CMK was found to correlate with its redundant inhibitory effect on the TL activity of proteasomes, suggesting that furin inhibition itself does not promote HBV replication. Furthermore, ETV combined with CMK was found to reduce HBV replication and HBeAg secretion simultaneously, which are concordant with the virological and serological responses pursued with a vengeance in antiviral practice.

HBV replication enhancements of CMK and the mutation (T147A) are accompanied by intracellular HBcAg accumulation [16], and HBcAg is structural protein with its intracellular level usually correlating with viral replication level [18, 19]. The findings support that HBcAg accumulation accounts for the HBV replication enhancement of CMK and the mutation. Because HBcAg and pre-HBe have identical furin-sensitive arginine-rich domains, the above findings also suggest that furin may proteolyze HBcAg. Therefore, it is reasonable to concern the unfavorable HBV replication-enhancing effect of furin inhibition. Fortunately, the suppression of furin itself was not found to enhance HBV replication in this study, which was evidenced by the facts: (i) D6R incubation and furin inhibitory prosegment expression inhibited HBeAg secretion, but did not enhance HBV replication; (ii) CMK enhanced HBV replication, but which was via a redundant inhibitory effect on the TL activity of proteasomes.

As furin is involved in the maturation of membrane fusion proteins and pro-toxins of bacteria and viruses, furin inhibition using inhibitors is viewed as a potential therapeutic strategy for anthrax, influenza A and Ebola virus infection [13, 29, 30].The significance of HBeAg to persistent HBV infection [8], the correlations of furin activity with the outcomes of HBV infection [14], and the lack of influences on cell secretory function [15], and HBV replication in this study suggest that the suppression of furin is also a promising novel strategy for the antiviral therapy of HBV infection in the future. Due to the lack of natural inhibitors, furin inhibitors used at present all are man-made compound. Although many researchers make effort to develop new inhibitors [24, 29, 30], no medicinal inhibitors are available at present. CMK and D6R are small synthetic inhibitors suitable for clinical purpose. CMK has used as a reference furin inhibitor in cell-based tests and inhibits HBeAg secretion in HepG2.2.15 cells [15, 16]; however, it enhances HBV replication, which is in conflict with the goal of antiviral therapy. Compared with CMK, D6R is more sensitive in cell-free test system or targeting furin on the cell surface, but less effective in inhibiting HBeAg secretion in cell-based test system [15, 24, 31, 32], perhaps due to its poor permeability [24]. For these reasons, the development of new furin inhibitors is imperative in the future. Our findings, including those in this study, are helpful to determine the target and orientation of the development process, paying attention to the specificity, especially not to affect the TL activity of proteasomes, and the permeability to reach the *trans*-Golgi network.

Since the successful development of new inhibitors of furin has a long way to go, to make use of current inhibitors is a reasonable option. In this study, ETV was found to abrogate the HBV replication enhancement of CMK, and ETV combined with CMK inhibited HBV replication and HBeAg secretion simultaneously in HepG2.2.15 cells. Although no synergistic effect was observed, these results imply that it would be possible to realize the virological and serological responses of the antiviral therapy rapidly and economically. However, much more studies should be carried out to clarify the effectiveness and safety of the combination strategy *in vivo*. Theoretically, ETV abrogates HBV replication enhancement but HBcAg accumulation of CMK. The remained HBcAg accumulation may have a chance to induce the aggravation of the inflammation and cell injury by increasing the presentation of HBcAg epitopes. It is less likely, however, to lead to fatal liver dysfunction since the generation of these epitopes depends on cellular proteasomes that have been inhibited by CMK. Of course, new effective inhibitors without effects on HBcAg accumulation would be the first choice of the combination therapy in the future. Besides HBeAg, CMK and furin knockdown with small interfering RNA lead to the reduction of HBsAg [33]. Our previous study further demonstrates that CMK only at higher concentration (100 μmol/L) suppresses the biosynthesis of HBsAg [15]. In this study, HBsAg-reducing effect of CMK was not found, perhaps due to the lower concentration (20 μmol/L) employed. Nonetheless, it is important to pursue the inhibitory effect on HBsAg in novel inhibitor development or therapy regimen establishment in the future since HBsAg seroconversion is the perfect end-point of antiviral therapy for chronic HBV infection.

## Conclusions

Furin-inhibiting measures other than CMK did not enhance HBV replication and CMK enhanced HBV replication by affecting the TL activity of proteasomes, suggesting that furin inhibition itself does not lead to HBV replication. ETV could completely abrogate the HBV replication enhancement of CMK. Moreover, ETV combined with CMK reduced HBV replication and HBeAg secretion simultaneously in HepG2.2.15 cells, which implies that nucleotide/nucleoside analogs combined with some furin inhibitors may be an easy way to realize the dual goals of antiviral therapy. Nonetheless, more studies on the effectiveness and safety of the combination strategy *in vivo* are warranted in the future.

## Methods

### Plasmid construct

Furin inhibitory prosegment-expressing vector (pfurin-PS) was constructed using plasmid pIRES2-EGFP (Clontech, Palo Alto, CA). The sequence of the inhibitory prosegment was designed from those coding 109 amino acids of the N-terminus of furin (gene ID: 5045). The sequence of the construct had been confirmed using DNA sequencing.

### Cell culture, transfection, and protease inhibitor treatments

HepG2.2.15 cells were regularly grown in Dulbecco’s modified Eagle’s medium, supplemented with 10% (vol/vol) fetal calf serum and 380 μg/mL of geneticin if necessary. Transient transfection was performed using FuGENE HD transfection reagent (Roche Applied Science, Indianapolis, IN). Cells were treated with 10 ~ 50 μmol/L CMK (EMD Biosciences, La Jolla, CA, USA) or 100 μmol/L D6R (EMD Biosciences) with or without 30 nmol/L ETV (Sigma-Aldrich Corporation, St. Louis, MO, USA) for 48 hours in a growth arrest medium containing 0.5% (vol/vol) fetal calf serum after confluent growth. The cells (10^7^) were harvested to evaluate HBV replication and viral antigen expression. To perform virion release and cell viability assays, cells were further cultivated using fresh medium for 12 hours. To evaluate the turnover rate of HBcAg, cells were treated with or without cycloheximide, a protein synthesis inhibitor (Sigma-Aldrich Corporation, St. Louis, MO, USA), and harvested in 12 hour intervals to a maximum of 48 hours.

### Detections of core-associated HBV DNA

The isolation of supernatant and intracellular core particles was performed as reported [34]. Sampling was balanced based on the protein level in cell lysate. Supernatant core-associated HBV DNA was quantitatively analyzed using commercial real-time fluorescent polymerase chain reaction (PCR) kits (Daan Gene Inc., Guangzhou, China). The intracellular core-associated HBV DNA was detected used Southern blot analysis. The isolated DNA was separated and transferred onto nylon membranes (Roche Applied Science, Indianapolis, IN, USA). After hybridized with digoxigenin-labeled DNA probes, all membranes were incubated with horseradish peroxidase-labeled anti-digoxigenin antibody (Roche Applied Science), and developed with an enhanced chemiluminescence reagent (Invitrogen Corporation, Shanghai, China).

### Detections of intracellular viral antigens, furin inhibitory prosegment and proteasome subunits

For the detections of proteasome subunits or intercellular HBeAg, pre-HBe and HBcAg, total cellular protein or cytosolic and non-cytosolic cellular proteins were extracted as reported [20]. The total or sorted cellular proteins were separated and transferred onto polyvinylidene fluoride membranes (Millipore Corporation, Billerica, MA, USA) using standard techniques. Immunoblot analysis was performed using polyclonal antibodies to HBcAg (DAKO, Carpinteria, CA, USA), furin (LS-C23720; LifeSpan BioSciences Inc., Seattle, WA, USA) or the proteasome subunits (ab22673; Abcam, Cambridge, UK).

### Protease digestion assay

The trypsin (Sigma-Aldrich Corporation) digestion of recombinant HBcAg, a fragment of 156 amino acids with a 6 × histidine (his)-tag in the C-terminus (Millipore Corporation), was performed as described elsewhere [22]. To study the putative proteolysis of recombinant HBc by the cytosolic proteases of cells, cytosolic extract was prepared from 10^6^ HepG2 cells by incubating for 3 minutes on ice with 1 mL of a digitonin buffer (50 mmol/L Tris [pH 8], 150 mmol/L NaCl, and 22.5 μg/mL digitonin), and centrifuging at 1500 × g for 2 minutes at 4°C.

### Proteasome activity assay

The TL, chymotrypsin-like, and caspase-like activities of the proteasomes were measured using commercial cell-based kits (Proteasome-Glo^TM^, Promega, Madison, WI, USA) as described previously [35]. Luminescence was recorded using a luminometer (Promega).

### Statistical analysis

The differences in virion release, HBeAg and HBsAg secretions and proteasome activities were analyzed using the Student’s *t*-test. A *P* < 0.05 was considered statistically significant. All statistical analyses were conducted using SPSS software (version 11; SPSS, Inc., Chicago, IL, USA).

## Abbreviations

chTL: Chymotrypsin-like
CMK: Decanoyl-RVKR-chloromethylketone
D6R: Hexa-D-arginine
ETV: Entecavir
HBcAg: Hepatitis B core antigen
HBeAg: Hepatitis B e antigen
HBsAg: Hepatitis B surface antigen
HBV: Hepatitis B virus
pre-HBe: Hepatitis B e antigen precursor
PCR: Polymerase chain reaction
TL: Trypsin-like.
